# Infertility in the Moroccan population: an etiological study in the reproductive health centre in Rabat

**DOI:** 10.11604/pamj.2018.30.204.13498

**Published:** 2018-07-10

**Authors:** Amal Benbella, Siham Aboulmakarim, Houyam Hardizi, Asmaa Zaidouni, Rachid Bezad

**Affiliations:** 1 Assisted Reproductive Technology Unit of the Reproductive Health Centre, University Hospital Ibn Sina, Rabat, Morocco; 2 Laboratory of Medical Biotechnology (Med Biotech), Faculty of Medicine and Pharmacy, University Mohamed V, Rabat, Morocco

**Keywords:** Primary infertility, male infertility, female infertility

## Abstract

The causes of infertility vary widely and differ between regions and within countries. There is no report on this subject in Morocco. Therefore, the aim of this study was to determine the causes of infertility in Moroccan infertile couples and to compare the findings with data from the various published studies. This retrospective study included 1265 infertile couples who attended the Assisted Reproductive Technology Unit of the Reproductive Health Centre of the University Hospital Ibn Sina in Rabat. All couples had been infertile for at least 1 year and both partners were fully investigated. The median duration of infertility was 5 ± 4 years. Couples had primary and secondary infertility in 77.2% and 22.8% of cases, respectively. Among the 1265 couples, 39.6% had a female factor, 28.2% had a male factor, 17% had both male and female factors and in 15.2% of couples, the cause of infertility was undetermined. The most common causes of male infertility were varicocele (14.3%), obstructive azoospermia (7%), Congenital anomalies (5.5%) and male accessory gland infection (4%). Results showed that 54.8% of men had a normal semen analysis. Among women, infertility factors were ovulatory disorders (27.5%), tubal factor (26.6%), uterine factor (12.6%), endometriosis (4.1%), and 43.4% of women were normal. The causes of infertility in this study are comparable with those reported by the World Health Organization and other studies. However, the substantial delay before attending an infertility clinic highlighted by the study needs additional consideration.

## Introduction

Infertility, defined as “the failure to achieve a clinical pregnancy after 12 months or more of regular unprotected sexual intercourse“, does not only have health implications for those involved, but is a condition linked with individual human rights [1, 2]. Nearly 72.4 million couples suffer from reproductive disorders [3]. However, infertility rates differ between countries ranging from 5 to 8% in developed countries and from 5.8% to 44.2% in developing countries [4]. Variations are also noticed within the same country: Iran 3 to 8%; United Kingdom 2 to 26%; and China 1 to 18% [4]. Infertility's extensive psychosocial and economic impact on its sufferers, which is recognized worldwide, is more significant in developing countries [1, 5]. In fact, infertile couples in these regions, in addition to distress and personal devastation, experience more social stigmatization, exclusion, marital problems (divorce or polygamy), and economic challenges due to the cost of assisted reproductive technologies [1, 2]. The most effective approach to solving the infertility problem is, in addition to prevention and education, the implementation of appropriate infertility treatments [6]. Therefore, precise information on the causes of infertility is essential for reproductive health care providers as well as policymakers [2]. The causes of infertility vary widely and differ between regions and within countries [7, 8]. This discrepancy is due to the existence of differences in cultural, socioeconomic, health care practices and policies, and environmental conditions [6, 8]. Although many studies have investigated the causes of infertility worldwide, there is no comprehensive research on the subject in Morocco. Thereby, the objective of the present study was to determine the etiologic spectrum of infertility in the Assisted Reproductive Technology Unit of the Reproductive Health Centre of the University Hospital Ibn Sina in Rabat, and to compare the results with data from the various published studies.

## Methods

In this retrospective study the medical records of 1265 infertile couples attending the Assisted Reproductive Technology Unit of the Reproductive Health Centre of the University Hospital Ibn Sina in Rabat, from October 2013 to March 2017, were reviewed. The complete history of all patients was detailed and a complete physical examination was conducted. Only couples who had been infertile for at least 1 year, whose investigation had been completed and of whom both partners had received a diagnosis, were admitted to the study. For men, the history of pubertal development, cryptorchidism, inguinal surgery, orchitis, testicular torsion, sexually transmitted diseases, drugs or hormones intake as well as a history of exposure to chemicals were documented. The physical examination evaluated the size and position of the testes and the presence of varicocele and any other congenital or acquired anomalies. For women, a particular attention was paid to the pattern of pubertal development, menstrual history, abdominal and pelvic surgery, drugs or hormone intake and sexually transmitted diseases. Sexual history focused on libido, erectile function, frequency and timing of intercourse. A detailed gynecologic examination was performed to ascertain congenital or acquired anomalies or diseases such as endometriosis. Specific investigations were carried out for the male partner including semen analysis and semen culture. Hormone analysis, ultrasonography and Doppler ultrasound of the scrotum were performed in some cases. Semen analysis was done according to the World Health Organization laboratory manual. For the female partner, the specific investigations performed included hormone analysis, ultrasonography and hysterosalpinography for tubal patency assessment. Hysteroscopy was performed in some cases. Laparoscopy was done when indicated to study tubal anomalies and to look for endometriosis. Statistical analysis was performed using the software package SPSS 20 (IBM) (Statistical Package for the Social Sciences). The study was approved by the Ethics Committee of Biomedical Research of the University Mohammed V of Rabat, Morocco.

## Results

One thousand two hundred and sixty five infertile couples were assessed in this study. Of these, 77.2% had primary infertility and 22.8% had secondary infertility. The median age of men was 39 ± 7 years with extreme ages of 21 and 67 years, and the median age of women was 32 ± 5.4 with extreme ages of 18 and 45 years. Most women were between the ages of 25-30 years (29.3%) and 35-40 years (29.8%) while most men were between the ages of 35-40 years (34.8%). The median duration of infertility at the time of the first medical consultation in the infertility unit was 5 ± 4 years with extreme duration of 1 and 24 years (Table 1). All 1265 men selected in this study had at least two semen analysis. Among these men, 54.8% had a normal semen analysis and 45.2% had abnormal seminal parameters. The most frequent abnormalities were oligoasthenoteratospermia (11%) and Azoospermia (9.3%) (Table 2). Table 3 and Table 4 give the distribution of the causes of infertility in the male and the female partners. Varicocele associated with an abnormal semen analysis was the most frequent cause of male infertility observed in 14.3% of cases. However, 33 men had a varicocele without any effect on semen quality. Obstructive azoospermia was detected in 7% of cases. Despite a complete investigation, there were 156 male partner with abnormal semen analysis, without any recognizable cause found in their medical history, physical examination and hormone assay. Among women with a diagnosed cause of infertility, 27.5% had diagnoses attributable to ovulatory disorders, which represented the second most common etiologic factor of infertility identified in the study couples. Polycystic ovary syndrome was found in 18.3% of women. In addition, there were 12 patients with clinical and biochemical features of premature ovarian failure. The other main cause of infertility was tubal factors diagnosed in 26.6% of women. There were 12.6% of women with uterine factors. Endometriosis was observed in 4.1% of cases. Despite a full set of investigations, 549 (43.4%) women had no demonstrable cause of infertility while 162 (12.8%) women had more than one cause of infertility. In fact, the total number of diagnoses of infertility made (909) was higher than the number of infertile women diagnosed (716). Thus, 554 (43.8%) women had one cause of infertility, 148 (11.7%) women had two causes, and 14 (1.1%) women had three causes of infertility. The investigations of the study couples revealed that 501 (39.6%) of them had infertility due to a female factor only, 357 (28.2%) had infertility due to a male factor only and in 192 (15.2%) cases, the cause remained unexplained. In 215 (17%) cases, a male as well as a female factor were identified.

**Table 1:** Characteristics of the study couples

Characteristics	Number of cases	Percentage %
**Female partner age**		
≤24 years	135	10.7
25-30 years	371	29.3
31-34 years	335	26.5
35-40 years	377	29.8
>40 years	47	3.7
**Male partner age**		
≤24 years	8	0.6
25-30 years	121	9.6
31-34 years	180	14.2
35-40 years	440	34.8
41-44 years	226	17.9
45-50 years	205	16.2
>50 years	85	6.7
**Type of infertility**		
Primary	977	77.2
Secondary	288	22.8
**Duration of infertility**		
≤2 years	225	17.8
3-5 years	492	38.9
6-7 years	218	17.2
≥8 years	330	26.1

**Table 2:** Distribution of semen abnormalities among men

Diagnosis	Number of cases identified	% of cases identified
OligoAsthenoTeratospermia	139	11
Azoospermia	118	9.3
Asthénospremia	85	6.7
OligoAsthénospermia	77	6.1
AsthenoTeratospermia	63	5
Tératospermia	43	3.4
Oligospermia	37	2.9
OligoTeratospermia	4	0.3
No semen	6	0.5
Normal semen	693	54.8

**Table 3:** Distribution of the causes of infertility among men

Male Diagnoses	Number of cases identified	% of cases identified
No demonstrable cause	693	54.8
Varicocele	180	14.3
Idiopathic sperm abnormalities	156	12.3
Obstructive azoospermia	89	7
Congenital anomalies in urogenital tract	69	5.5
Male accessory gland infection	51	4
Endocrine causes	8	0.6
Sexual dysfunction	6	0.5
Acquired testicular damage	5	0.4
Genetic causes	5	0.4
Immunological factors	3	0.2

**Table 4:** Distribution of the causes of infertility among women

Diagnosis	Number of cases identified	% of cases identified
**Ovulatory disorders**	349	27.5
Polycystic Ovary Syndrome	231	18.3
Ovulation dysfunction	75	5.9
Hyperprolactinemia	25	2
Premature Ovarian Failure	12	0.9
Hypogonadotropic Hypogonadism	6	0.5
**Tubal factor**	337	26.6
**Acquired Uterine abnormalities**	127	10
**Congenital abnormalities**	33	2.6
**Endometriosis**	52	4.1
**Endocrine causes**	11	0.9

## Discussion

Infertility is a global medico-socio-cultural problem whose prevalence remains a subject to controversy [2]. In fact, the prevalence of infertility differs between countries [2]. In the United Kingdom and the Middle East, 10 to 15% of couples have a difficulty to conceive [3]. This situation concerns almost one third of couples in the central and southern parts of Africa [3]. However, in other parts of the world, a decline in the incidence of infertility has been reported. For instance, in the United States of America it is estimated to 9% (range: 3.5% - 16.7%) and the prevalence of infertility is considered to be almost 8.5% in Canada [3]. Primary infertility is when no conception has ever occurred despite 12 months or more of regular unprotected sexual intercourse, whereas, secondary infertility is when a previous pregnancy was obtained but the couple is subsequently unable to conceive despite 12 months or more of regular unprotected sexual intercourse [1]. In the case of breastfeeding, exposure to pregnancy starts from the beginning of regular menstruation following delivery [1]. In this study, primary infertility was observed in 77.2% of couples. This relatively high frequency of primary infertility was also reported by Parsanezhad's meta-analysis in Iran where primary infertility was documented in 78.4% of couples [3]. A World Health Organization (WHO) study undertaken in thirty-three medical centres in twenty-five developed and developing countries found that most infertile couples around the world suffer from primary infertility [7]. However, a study in Nigeria reported a secondary infertility rate up to 85.7% of infertile couples [9]. This result confirms the WHO report, which stated that in Sub-Saharan Africa most couples (52%) suffered from secondary infertility [7]. Relatively high rates of secondary infertility (40%) are also observed in Latin America [7]. Asia, on the other hand, is different with only 23% of infertile couples suffering from secondary infertility [7]. A study conducted in Thailand confirmed that trend with 61.8% of primary infertility and 35.6% of secondary infertility [10]. In contrast, in Mongolia, secondary infertility was reported in 43.7% of couples [11]. The high rate of secondary infertility in these regions is mainly due to post-abortal and puerperal infections [2]. The woman's age and the duration of infertility have a major impact on fertility rates [5]. Indeed, the age of the woman influence strongly the chances of pregnancy [12]. It also represent one of the key parameters predicting the ovarian response to stimulation and the probability of a successful pregnancy during assisted reproductive technologies [12]. After the age of 35 years, pregnancy rates start to decrease in an accelerated downward trend [13]. This relation between the age and the decline in female fertility is independent from the male factor [13].

According to the WHO study, 25% of infertile women in developed countries were 24 years old or younger while this age range represented 22% to 42% of infertile women in developing countries [7]. In addition, in Orhue's study, 50% of infertile women were younger than 30 years old [9]. In contrast, in our study, only 10.7% of the women were 24 years old or younger while 33.5% of them were aged 35 years or older. Women's age in the present study is, indeed, older than in other studies conducted in developing countries. This could be explained by the delay in childbearing due to a late marriage, the pursuit of a career or financial stability, and the unawareness of the influence of age on fertility. This lack of accurate knowledge regarding infertility as well as the high cost of assisted reproductive technologies in Morocco are the main reasons for the prolonged duration of infertility found in this study. The study findings regarding the duration of infertility were similar to other results from other developing countries [5, 6, 8, 11, 14]. The length of time during which the study couples have been attempting to conceive was 8 years or more in 26.1% of cases whereas in developed countries only 7% are infertile for 8 years or more [5, 7]. In fact, at the time of the first medical consultation in an infertility unit, half the couples in the developed regions have a duration of infertility of less than 2 years while over two-thirds of couples in developing areas have a duration of infertility of more than 2 to 5 years [7]. In this study, only 17.8% of the couples requested medical care within 2 years of trying to conceive. There are four categories of infertility causes: female factor only, male factor only, combined factors and unexplained infertility [6]. According to the WHO study, the female factor is the most identified cause of infertility in all regions around the world when compared to the male factor, and this female/male difference is the highest in Africa [7]. However, data from various other studies are heterogeneous. In some studies, like in the present one, the female factor was the main cause of infertility [5, 7, 9, 11]. For example, in a multicenter survey conducted in three regions in France, female factors were responsible for infertility in 30% of couples while the male factors were found in 20% of cases [15]. The same trend was observed in Larsen's study in Tanzania, with 65.9% of female factors and 8.8% of male factors [16]. This high rate of female factors was also reported by Philipov et al in Western Siberia, where the women alone were responsible for infertility in 52.7% of cases and the man alone in 6.4% of cases [17]. In contrast, in other studies the male factor was found to be the most important etiological factor [6, 10, 14] (Table 5). This relatively wide difference between studies may be due to the diversity of the conditions involved in infertility [8].

**Table 5:** Studies describing the causes of infertility by gender in infertile couples

Study (Author, year, country)	Total infertile couples	Female factor (%)	Male factor (%)	Combined factors (%)	Unexplained (%)
Thonneau P, 1991, France [15]	1686	30	20	39	8
Zargar AH, 1997 Kashmir India [5]	250	57.6	22.4	5.2	14.8
Philippov OS, 1998, Western Siberia[17]	168	52.7	6.4	38.7	2.2
Bayasgalan G, 2004, Mongolia [11]	430	45.8	25.6	18.8	9.8
Larsen U, 2005, Tanzania [16]	91	65.9	8.8	13.2	12.1
Kamali M, 2007, Iran [14]	2492	28.6	50.5	11.6	9.3
Orhue A, 2008, Nigeria [9]	1948	34	18	28	20
Chiamchanya C, 2008, Thailand [10]	1072	17.5	19.4	55.6	4.7
Malekshah AK, 2011, Iran [6]	3734	34.7	38.9	14.6	11.8
The present study	1265	39.6	28.2	17	15.2

In most studies conducted in other countries, male factor has been identified in approximately 50% of infertile couples [18]. In the present study, a male factor was found in 45.2% of couples and the causes of male infertility showed a comparable spectrum of etiologies as reported by other studies [6, 7, 11, 18, 19]. Varicocele was the main diagnosis observed. Similarly, varicocele was found in 42.7% of cases in Malekshah's study in Iran [6], and in 75.7% of cases in Jeje's study in Nigeria [1]. Varicocele is the most common potentially treatable genital disease in infertile men [18]. Its prevalence has been estimated to 15-20% in general population and 30-40% in infertility clinic's patients [18]. However, in the literature, the role of varicocele as a causal factor of male infertility remains a subject to controversy [18]. In fact, despite a reported improvement in semen quality after varicocele ligation, the randomized controlled trials were inconclusive with respect to pregnancy rates [19]. In a monocenter study published by a tertiary referral andrology center in Rotterdam, 44% of the study population had unexplained impaired semen quality [19]. Similar results were reported by other studies [7, 11]. In our study, the cause of seminal abnormalities remained unknown in 12.3% of the study men. These findings reveals an evident gap in our current comprehension of the causes, biological mechanisms and pathways of spermatogenesis alterations and male reproductive physiology [18]. Concerning the other causes of male infertility documented in the present study, results were heterogeneous when compared to other reports. For example, in the study conducted by Pierik et al, immunological infertility represented 11% of cases followed by accessory gland infection (5.3%), cryptorchidism (9.0%), sexual dysfunction (4.6%), acquired testicular factor (4.1%), hypogonadotropic hypogonadism (3.4%) and obstructive azoospermia (1.1%), while genetic factors had not been analyzed [19]. In a study in the State Research Center on Maternal Child Health of Ulaanbaatar, Mongolia, obstructive azoospermia was found in 8.4% of cases followed by male accessory gland infection (6.7%), acquired testicular damage (5.3%), endocrine causes (4.6%), and congenital anomalies in urogenital tract (2.8%). There was no case of sexual dysfunction or immunological factors [11]. Diagnoses attributable to ovulatory disorders in women were the second most common etiologic factor of infertility identified in our study (27.5%). Similar results were reported in Iran (28.8%) [6]. In fact, in Malekshah's study, polycystic ovary syndrome was the main ovulatory disorder documented in 56.8% of women having an ovulatory disorder [6].

Likewise, a polycystic ovary syndrome was found in 66.2% of women having an ovulatory disorder in the present study. The first line of treatment of polycystic ovary syndrome remains ovulation induction. This makes it one of the causes of infertility whose treatment is within the reach of the general population, especially in countries where accessibility to assisted reproductive technologies, like in Morocco, remains hampered by their high cost. However, in our study only 9.4% of women had polycystic ovary syndrome as the sole cause of infertility. In contrast, premature ovarian failure, which was observed in 0.9% of the study women, represents the end of all therapeutic possibilities in Morocco. In a country where oocyte donation is not allowed this diagnosis is dread by women as well as by reproductive health care providers. However, the ovarian tissue activation recently developed in Japan seems a promising new therapeutic approach for these cases. In Bayasgalan's study, premature ovarian failure was reported in 0.2% of cases while in other studies, it was not distinguished from the other ovulatory disorders and therefore not analyzed as a separate etiologic factor [11]. The other ovulatory disorders that could be treated without assisted reproductive technologies are hyperprolactinemia and hypogonadotropic hypogonadism, yet they were found in only 1.4% and 0.5% of cases respectively. According to the WHO study, the occurrence of hyperprolactinemia ranges from 5 to 8% in infertile women around the world [7]. In addition, Bayasgalan et al have reported hyperprolactinemia in 6.7% of cases and Zargar et al have found it in 8.4% of women [5, 11]. Among the female causes of infertility, tubal factor ranked second (26.6%). Similar findings were reported by other studies [5, 6]. In the WHO study, 11% of infertile women in developed countries had tubal occlusions while this etiologic factor was present in 49% of cases in Africa and in 14 to 20% of cases in other non African developing countries [7]. This high rate of tubal occlusion is mainly attributed to the high frequency of genital infections in these regions [7]. In our study, 82 (24.3%) women with a tubal factor had a history of tuberculosis, which is an endemic disease in Morocco. Endometriosis is “an estrogen-dependent chronic inflammatory condition that affects women in their reproductive period causing infertility and pelvic pain“ [20]. This disease induces an alteration in endometrial receptivity due to cytokine mediated inflammation; has a detrimental effect on ovarian physiology; and causes anatomic distortions and adhesions [20]. Thereby, it affects spontaneous fertility as well as assisted reproductive technology outcome. Endometriosis is most common in Asian women (10%), followed by those in developed countries (6%) [7]. Indeed, in Thailand, endometriosis was found in 25.6% of infertile women while in Mongolia, it was reported in only 4.2% of cases [10, 11]. In the present study, it was diagnosed in 4.1% of women. Although uterine factors are more incriminated in recurrent pregnancy loss and preterm delivery, they are also reported as a cause of infertility [8]. In the present study, uterine causes were observed in 12.6% of women. However, the results of some studies are quite different. For example, uterine factors were reported in 9.1% of cases in Chiamchanya's study and in 16.7% of cases in Masoumi's study [8, 10]. Concerning Hypothyroidism which was detected in 0.9% of cases, it is known that its existence can impair fertility [5], yet few studies have reported that etiologic factor.

## Conclusion

Infertility is a real health problem affecting the individuals and the society. This study showed that infertility is due to a broad range of causes and that a considerable proportion of theses etiologies requires assisted reproductive technologies. In Morocco as in many other developing countries, assisted reproductive technologies are not available in all the regions and are still not affordable by the general population. This center in Rabat is the first public assisted reproductive technology unit in Morocco. Therefore, this study testifies to the need to implement other similar units in low-income countries in order to decrease the burden of infertility to its sufferers.

### What is known about this topic

- Infertility is the failure to achieve a clinical pregnancy after 12 months or more of regular unprotected sexual intercourse;
- 10 to 15% of couples have a difficulty to conceive;
- The causes of infertility differ between regions and within countries.

### What this study adds

- There is a high frequency of primary infertility in the studied population;
- There is a substantial delay before the first medical consultation of the infertile couples;
- 45.2% of infertile men had abnormal semen parameters.

## Competing interests

The authors declare no competing interests.
